# Estimating and modelling cure in population-based cancer studies within the framework of flexible parametric survival models

**DOI:** 10.1186/1471-2288-11-96

**Published:** 2011-06-22

**Authors:** Therese ML Andersson, Paul W Dickman, Sandra Eloranta, Paul C Lambert

**Affiliations:** 1 Department of Medical Epidemiology and Biostatistics, Karolinska Institutet, Box 281, 171 77 Stockholm, Sweden; 2 Department of Health Sciences, University of Leicester, Leicester, UK

## Abstract

**Background:**

When the mortality among a cancer patient group returns to the same level as in the general population, that is, the patients no longer experience excess mortality, the patients still alive are considered "statistically cured". Cure models can be used to estimate the cure proportion as well as the survival function of the "uncured". One limitation of parametric cure models is that the functional form of the survival of the "uncured" has to be specified. It can sometimes be hard to find a survival function flexible enough to fit the observed data, for example, when there is high excess hazard within a few months from diagnosis, which is common among older age groups. This has led to the exclusion of older age groups in population-based cancer studies using cure models.

**Methods:**

Here we have extended the flexible parametric survival model to incorporate cure as a special case to estimate the cure proportion and the survival of the "uncured". Flexible parametric survival models use splines to model the underlying hazard function, and therefore no parametric distribution has to be specified.

**Results:**

We have compared the fit from standard cure models to our flexible cure model, using data on colon cancer patients in Finland. This new method gives similar results to a standard cure model, when it is reliable, and better fit when the standard cure model gives biased estimates.

**Conclusions:**

Cure models within the framework of flexible parametric models enables cure modelling when standard models give biased estimates. These flexible cure models enable inclusion of older age groups and can give stage-specific estimates, which is not always possible from parametric cure models.

## Background

Patient survival, the time from diagnosis to death, is the most important single measure of cancer patient care (the diagnosis and treatment of cancer). Cancer patient survival is often measured using 5-year relative survival, the proportion of patients that would still be alive 5 years after diagnosis if the cancer (either directly or indirectly) was the only possible cause of death [1]. As cancer patient survival has improved for many cancer types, and many patients are cured of their disease, another important question is what proportion of patients are cured of their cancer.

For most cancers the relative survival will reach a plateau some years after diagnosis, indicating that the mortality among the patients still alive is the same as expected in the general population. This point is called the cure point and the patients still alive are considered "statistically cured". De Angelis *et al*. [2], Verdecchia *et al*. [3], Yu *et al*. [4] and Lambert *et al*. [5] have proposed cure models for population-based cancer studies that can be used to estimate the proportion of cancer patients that are "statistically cured". The models also give an estimate of the survival of those "uncured". These measures are of interest to patients, clinicians and policy makers, and can give valuable insights into temporal trends in cancer patient survival. One limitation of parametric cure models is that the functional form of the survival of the "uncured" has to be specified. It can sometimes be difficult to fit survival functions flexible enough to capture high excess hazard within a few months from diagnosis, which is common among older age groups. This has led to the exclusion of older age groups in population-based cancer studies using cure models [6]. In our experience the current models can also give biased estimates, or fail to converge, when the cure proportion is high (e.g. 80% and above). Yu *et al*. [4] have proposed the generalized gamma distribution, which make less distributional assumptions, but computational difficulties may arise. Lambert *et al*. [7] have proposed a finite mixture of Weibull distributions to add flexibility, but this adds to the complexity of deciding which model parameters are allowed to vary by covariates since there are 4 Weibull parameters to be modelled. Non-parametric or semi-parametric cure models have been suggested (e.g. [8-11]), but they do not use relative survival.

This paper shows how these problems could potentially be avoided by using flexible parametric survival models to estimate the cure proportion and the survival of the "uncured" in a population-based setting. Flexible parametric survival models were first introduced by Royston and Parmar [12,13], and extended to relative survival by Nelson *et al*. [14] and Lambert and Royston [15]. The models are fitted on the log cumulative excess hazard scale using restricted cubic splines for the baseline. By the use of splines these models can more easily capture the shape of the underlying distribution. We illustrate the method using data on patients diagnosed with colon cancer in Finland during 1953-2003, which has previously been used to study temporal trends in the cure proportion [6]. We use and further develop the flexible parametric survival model. Our results are compared to the previously published results by Lambert *et al*. [6]. Here we also include patients 80 and above at diagnosis, who were excluded in the paper by Lambert *et al*. [6], as well as analysing a subset of the cohort with localised cancer to evaluate how the method perform when the survival is high.

## Methods

### Relative survival

The method of choice for studying cancer patient survival in a population-based setting is relative survival, *R*(*t*) [1]. Relative survival is the observed (all-cause) survival, *S*(*t*), among the cancer patients divided by the expected survival, S*(*t*), in a hypothetical group in the general population that is comparable to the cancer patients with respect to age, sex, calendar year and possible other covariates. An advantage of relative survival is that it does not rely on classification of cause of death, which is known to be poorly reported [16]. As for cause-specific survival, relative survival measures the net survival. The net survival at a certain point in time is the proportion of patients who would have survived up to that point if the cancer of interest was the only possible cause of death. Even though this measure might not be directly relevant from a patient perspective, it is useful for studying temporal trends in cancer patient survival and comparing populations where expected survival may vary. In the relative survival model the overall survival can be written as(1)

The hazard analogue of relative survival is the excess hazard rate. The overall hazard, *h*(*t*), among the patients is the sum of two components, the expected hazard, h*(*t*), and the excess hazard, *λ*(*t*), associated with a diagnosis of the cancer.(2)

Both S*(*t*) and h*(*t*) are assumed known and are usually obtained from routine data sources (eg. national or regional life tables).

### Parametric cure models

For most cancers the mortality in the patient group will, after some years from diagnosis, return to the same level as in the general population, i.e *λ*(*t*) in equation (2) is equal to zero after some time point. This point is called the cure point and the patients still alive are considered "statistically cured". This is a population definition of cure and does not necessarily imply that all patients are medically cured. Statistical cure is a useful method of measuring long-time survival in population-based cancer studies. One of the most often used cure models in population-based cancer studies is the mixture cure model [2,3,5]. When incorporating relative survival, the overall survival function from the mixture cure model can be written as(3)

It assumes that a proportion, *π*, of the patients will be cured (do not experience excess mortality), while the remainder, 1 *- π*, are "uncured". *S_u _*(*t*) is the cancer-specific survival function for the "uncured", and is estimated by the model along with the cure proportion. A parametric distribution for *S_u_*(*t*) has to be chosen, and a Weibull distribution is often used [2,3,5,6].

Another parametric cure model used in population-based cancer studies is the non-mixture cure model [5], which estimates an asymptote for the survival function at the cure proportion. The survival function for the non-mixture model can be written as(4)

where *F_Z_*(*t*) is a distribution function, as for the mixture model, a Weibull distribution is often used. The non-mixture model can be written as a mixture model(5)

which enables estimation of both the cure proportion and the survival of the "uncured". When modelling, both the cure proportion and the parameters in *S_u_*(*t*) or *F_Z_*(*t*) can be allowed to vary by covariates.

### Flexible parametric survival model

The flexible parametric survival model [14,15] is fitted on the log cumulative excess hazard scale, using restricted cubic splines to estimate the baseline cumulative excess hazard. By integrating equation (2) we get(6)

where *H*(*t*) is the overall cumulative hazard, H*(*t*) the expected cumulative hazard and Λ(*t*) is the cumulative excess hazard. The reason for modelling on the log cumulative excess hazard scale instead of the log excess hazard scale is because the log cumulative excess hazard is a relatively stable function, and it is easier to capture its shape. We are interested in modelling the cumulative excess hazard on the log scale(7)

where *x *= ln(*t*) and *s*(*x*; ***γ*_0_**) is a restricted cubic spline function, defined as(8)

where *K *is the number of knots and the *j^th ^*basis function is defined as *v*_1_(*x*) = *x*, and for *j *= 2, ...*, K - *1 as(9)

where *u*_+ _= *u *if *u >*0 and *u*_+ _= 0 if *u ≤ *0, *k*_1 _is the position of the first knot, *k_K _*the position of the last knot, and . Up to the first knot, all spline variables except the first one (*v*_1_, the linear variable) are zero, so the log cumulative excess hazard is forced to be linear before first knot position. Introducing covariates, **z**, into equation (7) gives(10)

This is a proportional excess hazards model. Non-proportional excess hazards models, i.e. models with time-dependent covariate effects, are extremely common in population-based cancer studies and can be modeled by including interactions between covariates and splines for time. Since the time-dependent effects usually do not require as many knots as the baseline cumulative excess hazard, new spline parameters are introduced for each time-dependent effect, and separate knot positions can be chosen for each new covariate with a time-dependent effect, *z_i_*. This gives the model:(11)

where *D *is the number of time-dependent covariate effects and *s*(*x*; ***γ*_*i*_**) is the spline function for the *i^th ^*time-dependent effect.

### Flexible parametric cure models

When cure is reached the excess hazard rate is zero, and the cumulative excess hazard will be constant after this time. By forcing the log cumulative excess hazard in the flexible parametric survival model to not only be linear but also to have zero slope after the last knot, we would be able to estimate the cure proportion. This is done by calculating the spline variables "backwards", treating the knots in reversed order, and then restricting the linear spline variable to be zero. The spline basis functions, *v_j_*(*x*), are then defined as(12)

where *j *= 2, ..., *K *-1, and  The relative survival function from the flexible parametric survival model, with splines calculated backwards and with restriction on the parameter for the linear spline variable (*γ*_01 _= 0) is defined as(13)

which can be written as(14)

where *π *= exp(- exp(*γ*_00_)). When comparing to a non-mixture model we can see that the flexible parametric cure model is a special case of a non-mixture cure model with *π *= exp(- exp(*γ*_00_)), and *F*_*Z*_(*t*) = exp(*γ*_02_*v*_2_(*x*) + ... + *γ*_0*K *- 1_*v_K - 1_*(*x*)). *F_Z_*(*t*) is a distribution function as long as the excess mortality is not negative, which is very uncommon. As for the non-mixture model, the flexible parametric cure model can be written as a proportional excess hazards model, as long as no time-dependent effects are modelled. When we incorporate covariates,(15)

we see that the constant parameters, *γ*_00 _and ***β ***are used to model the cure proportion and the time-dependent parameters are used to model the distribution function *F_Z_*(*t*). The constraint of a zero effect for the linear spline term has to be incorporated for each spline function, *s*(*x*; ***γ**_**i**_*), that we model. All spline variables take the value 0 from the point of the last knot, which means that in equation (15), the constant parameter, *γ*_00_, is the log cumulative excess hazard at and beyond the last knot for the reference group, and can therefore be used to predict cure. It is usually preferred to orthogonalise, i.e. by Gram-Schmidt orthogonalisation, the spline variables. This results in them not being zero from the point of the last knot, and cure can then not be predicted by a direct transformation of the constant parameters. Therefore, we have chosen to center the orthogonalised spline variables around the value they take at the last knot, which enables direct predictions of cure from the constant parameters. All parameters are estimated using maximum likelihood estimation on individual level data [15]. The survival of "uncured" can be predicted in the same way as for the non-mixture cure model, and the median survival time of "uncured" is predicted using a Newton-Raphson algorithm in a similar way as Lambert *et al*. [7].

We have adapted the Stata package for flexible parametric survival models [15], to incorporate backward calculation of the splines and the constraint to force a constant cumulative excess hazard after the last knot. There are also postestimation commands to predict the cure proportion and the survival of the "uncured".

### Evaluating the method

To evaluate the model we used data from the Finnish Cancer Registry. The Finnish Cancer Registry started in 1953, and the completeness for solid tumors is over 99% [17]. We studied all patients diagnosed with colon adenocarcinoma in Finland 1953-2003, with follow-up until 2004. Patients that emigrated were censored at the date of emigration, and everyone still alive was censored 10 years after diagnosis. Patients that were incidentally diagnosed at autopsy or were registered solely on death certificate information were excluded. The cohort consists of 34,664 patients. The same cohort, restricted to patients aged less than 80 years at diagnosis, is described elsewhere [6]. In that study, temporal trends of the cure proportion and the median survival time of uncured were estimated for different age groups, and we have repeated that analysis with the flexible parametric cure model.

We graphically compared the estimated relative survival from the flexible parametric cure model with empirical life table estimates of relative survival using the Ederer II [18] method. For comparison with the life table estimates the data were divided into 5 age groups (less than 50 years, 50-59 years, 60-69 years, 70-79 years and 80 years and above) and 5 calendar periods (1953-1964,1965-1974, 1975-1984, 1985-1994, 1995-2003).

Results from the flexible parametric cure model were also compared to results from a non-mixture cure model. Lambert *et al*. [6] used a mixture cure model with a Weibull distribution to study temporal trends by age group in survival of colon cancer patients in Finland. In that study, calendar year was modeled continuously using splines and age was categorized in four categories (less than 50 years, 50-59 years, 60-69 years and 70-79 years). The two main effects of year and age as well as an interaction between age and the linear spline variable for year were included for all three model parameters (the cure proportion and the two Weibull parameters). In this paper we repeated the analysis using a non-mixture cure model because it is more comparable with the flexible parametric cure model, but the estimates from the mixture and non-mixture cure models are very similar. We also included the oldest age group (80 years and above) that was excluded by Lambert *et al*. [6].

## Results

### Evaluating the sensitivity to knot placement

The flexible parametric survival model has been shown to be robust to the number and location of the knots [14,15]. To evaluate the sensitivity to the location of the knots for the flexible parametric cure model we compared predicted survival from the new model using different knot positions with life table estimates of relative survival. This was done separately for all combinations of age group and calendar period described previously. We used 6 knots and first distributed them according to default settings recommended by Lambert and Royston [15], evenly distributed at centiles of the log of the observed death times (centile 0, 20, 40, 60, 80 and 100). Since most of the death times happen early in follow-up the default positions put a lot of knots in the beginning of follow-up, so we also assessed putting more knots towards the end of follow-up, first by distributing 5 knots evenly according to centiles and one extra at the 95th centile (knot at centiles 0, 25, 50, 75, 95 and 100), and by placing more knots towards the end (at centiles 0, 35, 65, 80, 95 and 100). We also investigated the possibility to put the last knot earlier than the last observed death time, at the 95th centile (knots at centiles 0, 35, 65, 75, 85 and 95). To be sure that the knots are placed more evenly according to actual follow-up time we also put the knots at follow-up years instead of centiles of log death times (at the first and last death time and follow-up years 3, 5, 7 and 8). Finally we put the last knot after the last observed follow-up time (the knots were located at centiles 0, 25, 50, 75, 95 of log death times and the last knot 12 years from diagnosis).

**Figure 1:**
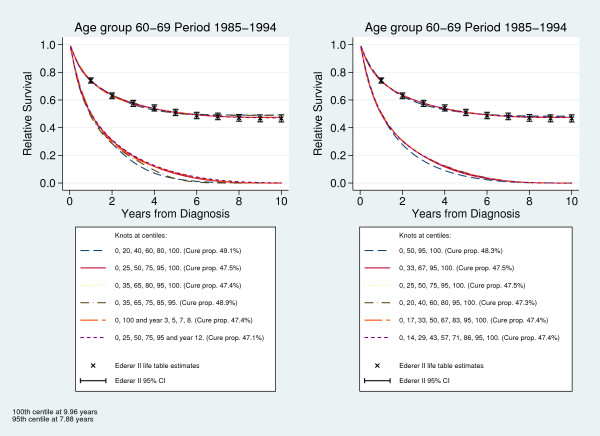
**Sensitivity to knot placement**. Ederer II life table estimates of relative survival and predicted survival from flexible parametric cure models with different knot locations and number of knots.

Similarly we evaluated the sensitivity to the number of knots used, ranging from 4 to 9 knots. The knots were distributed evenly according to centiles of log death times with an additional knot at the 95th centile. Figure 1 shows Ederer II life table estimates of relative survival and predicted relative survival as well as the survival of "uncured" from the flexible parametric cure models with the knot positions described above, for calendar period 1985-1994 and age group 60-69. The fit of the cure model is fairly robust to the number and location of the knots. When the default distribution is used all knots except the last one are placed within the first few years from diagnosis, and cure (where the survival reach a plateau) seems to be slightly overestimated (see Figure 1 left-hand panel). Cure is also slightly overestimated when the last knot is placed at the 95th centile of death times. The number of knots seem to have little impact on the estimated relative survival (see Figure 1 right-hand panel). The results also seem to be robust for the predicted survival function of the "uncured", with the largest differences being observed for the same combinations of knots that seem to overestimate cure. The results were similar for the other age groups and calendar periods (not shown). For almost all combinations of age group and calendar period the first or fourth knot distribution seem to overestimate the cure proportion, as compared to life table estimates. Similarly, for the different number of knots it was always the model with only 4 knots that gave slightly overestimated cure proportions. Among the other knot distributions the largest difference in the predicted cure proportion was observed for the age group 70-79 in period 1965-1974 (difference 2.2%) when the knots were placed at different locations, and for the oldest age group in period 1985-1994 (2.8%) when different number of knots were used. Overall the flexible parametric cure model seem to give a good fit as long as knots are placed over the whole follow-up period and the last knot is positioned at the last observed death time or possibly later.

### Comparison to life table and standard non-mixture model

**Figure 2:**
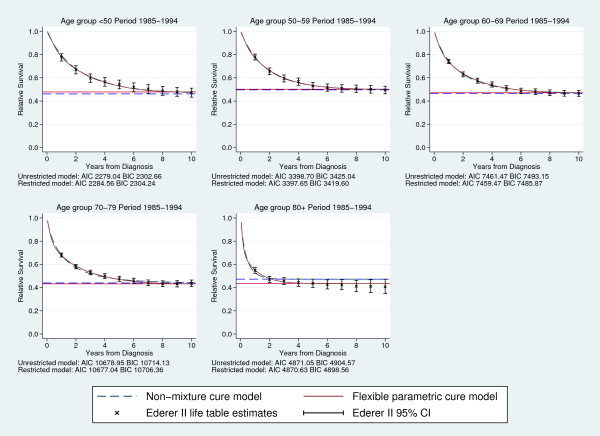
**Comparing predicted survival**. Predicted survival and cure proportions (%) from non-mixture models and flexible parametric cure models, compared to life table estimates of relative survival.

In order to see how well the flexible parametric cure model fitted the data we compared the predicted survival from a model with knots at centiles 0, 25, 50, 75, 95 and the last knot 12 years from diagnosis, with Ederer II life table estimates and the predicted survival from a non-mixture cure model using a Weibull distribution. This was done separately for all age groups and calendar periods. Figure 2 shows predicted survival from the two cure models, the predicted cure proportion and life table estimates for all age groups in calendar period 1985-1994. The predicted survival and cure proportions from the two cure models are similar for all age groups except the oldest, and they seem to correspond well with the life table estimates of relative survival. For the oldest age group, which was excluded in the paper by Lambert *et al*. [6] because of poor fit of the cure model used, the flexible parametric cure model fits the data much better than the Weibull non-mixture model (Figure 2).

All flexible parametric cure models described above were also compared to standard flexible parametric survival models, without the restriction of constant cumulative excess hazard after the last knot, using Akaike's information criterion (AIC) and the Bayesian information criterion (BIC). The difference between the models is then the restriction on the linear spline term to be zero. For most models the restricted 9 model gives a better fit, indicating that the final term in the unrestricted model is probably close to zero. In Figure 2 the AIC and BIC for all age groups in the calendar period 1985-1994 are shown. There is no formal goodness of fit test for cure models, since they rely on a good fit at the end of follow-up, and most of the data is at the beginning of follow-up. Since the flexible parametric cure model is a flexible parametric survival model with a restriction on one of the parameters, it can as described here, be compared to a standard flexible parametric survival model to test the assumption of cure. But we believe that this should not be used as a formal test, the assumption of cure and the fit of the model should be assessed graphically. To repeat and compare to the results from Lambert *et al*. [6] we also fitted a flexible parametric cure model and a non-mixture cure model. Calendar year was modeled continuously using splines and age was categorized in five categories as described previously. The two main effects of year and age as well as an interaction between age and the linear spline variable for year was included. All variables were included both as constant and time-varying effects, knots for the baseline log cumulative excess hazard were placed at centiles 0, 25, 50, 75, 95 and the last 12 years from diagnosis, and for the time-varying effects knots were placed at centiles 0, 25, 50, 75 and 100.

**Figure 3:**
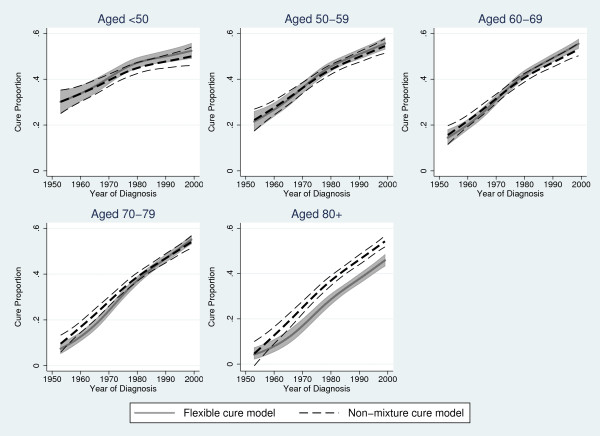
**Comparing predicted cure proportions**. Predicted cure proportion (%) with 95% confidence intervals from a flexible parametric cure model and a non-mixture cure model.

**Figure 4:**
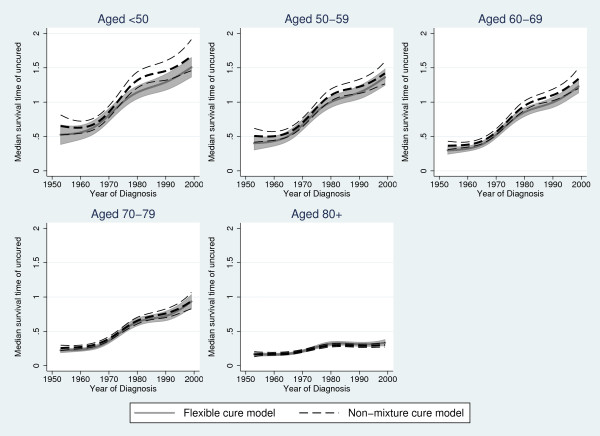
**Comparing predicted median survival times**. Predicted median survival time (years) of "uncured" with 95% confidence intervals from a flexible parametric cure model and a non-mixture cure model.

Table 1 shows predicted cure proportions from the flexible parametric cure model and the Weibull non-mixture model for year 1960, 1970, 1980, 1990 and 1999, and Table 2 the median survival time for "uncured" from the two models. In Figure 3 the estimated cure proportion from the flexible parametric cure model and the Weibull non-mixture cure model is shown and Figure 4 shows the estimated median survival time for "uncured" from the two models. The results from the two models are similar for most age groups, as expected. There are large differences between the estimated cure proportion from the two models for the oldest age group, where we know that the Weibull non-mixture model overestimates cure (Figure 2), and the flexible parametric cure model predicts a lower cure proportion for this age group. Some differences are also seen for the cure proportion among those aged 70-79 in earlier years, where we expect the non-mixture model to give slightly biased results [6].

**Table 1:** Estimates of cure

	Aged *<*50	Aged 50-59	Aged 60-69	Aged 70-79	Aged 80 and above
(a) Flexible parametric cure model					
Year 1960	33.8 (30.5-37.1)	26.7 (23.9-29.6)	20.2 (18.1-22.3)	12.7 (10.8-14.6)	7.9 (5.5-10.8)
Year 1970	40.7 (38.1-43.2)	36.2 (33.9-38.6)	30.9 (29.1-32.8)	24.1 (22.2-26.0)	16.9 (14.3-19.8)
Year 1980	47.2 (45.0-49.4)	45.6 (43.5-47.5)	42.0 (40.3-43.7)	37.2 (35.6-38.9)	28.6 (26.3-30.9)
Year 1990	50.0 (47.5-52.4)	51.0 (48.9-53.0)	49.3 (47.6-51.0)	46.9 (45.2-48.5)	37.7 (35.6-39.7)
Year 1999	52.4 (49.1-55.6)	55.6 (53.1-58.1)	55.5 (53.5-57.4)	55.0 (53.2-56.8)	45.8 (43.3-48.3)
(a) Non-mixture cure model					
Year 1960	33.7 (30.2-37.2)	27.6 (24.4-30.8)	21.8 (19.3-24.4)	16.9 (14.5-19.3)	12.7 (9.0-16.4)
Year 1970	39.5 (36.9-42.2)	36.2 (33.8-38.6)	31.5 (29.5-33.5)	28.1 (26.0-30.1)	25.0 (22.3-27.8)
Year 1980	44.9 (42.5-47.3)	44.3 (42.1-46.4)	40.7 (38.8-42.6)	38.8 (36.9-40.6)	36.9 (34.6-39.1)
Year 1990	47.7 (44.9-50.5)	49.8 (47.5-52.1)	47.3 (45.4-49.3)	46.9 (44.9-48.9)	46.2 (44.0-48.3)
Year 1999	50.0 (45.9-54.0)	54.5 (51.3-57.7)	53.0 (50.2-55.8)	54.0 (51.3-56.7)	54.2 (51.8-56.7)

Model based estimates of cure (%) from flexible parametric cure model and non-mixture cure model

**Table 2:** Estimates of median survival time of "uncured"

	Aged *<*50	Aged 50-59	Aged 60-69	Aged 70-79	Aged 80 and above
(b) Flexible parametric cure model					
Year 1960	0.56 (0.46-0.66)	0.43 (0.36-0.50)	0.32 (0.29-0.36)	0.23 (0.21-0.26)	0.16 (0.15-0.18)
Year 1970	0.81 (0.72-0.90)	0.67 (0.60-0.74)	0.53 (0.48-0.57)	0.36 (0.33-0.40)	0.21 (0.19-0.23)
Year 1980	1.14 (1.05-1.23)	1.00 (0.92-1.08)	0.85 (0.79-0.92)	0.64 (0.58-0.69)	0.32 (0.29-0.35)
Year 1990	1.29 (1.18-1.41)	1.14 (1.05-1.24)	1.00 (0.92-1.07)	0.72 (0.66-0.79)	0.31 (0.28-0.34)
Year 1999	1.51 (1.37-1.65)	1.36 (1.24-1.48)	1.23 (1.14-1.32)	0.93 (0.84-1.03)	0.33 (0.29-0.38)
(b) Non-mixture cure model					
Year 1960	0.63 (0.55-0.72)	0.50 (0.45-0.57)	0.38 (0.35-0.42)	0.27 (0.25-0.30)	0.17 (0.15-0.20)
Year 1970	0.86 (0.75-0.95)	0.70 (0.64-0.77)	0.56 (0.52-0.61)	0.39 (0.36-0.43)	0.21 (0.19-0.24)
Year 1980	1.32 (1.21-1.44)	1.10 (1.02-1.19)	0.95 (0.88-1.02)	0.66 (0.61-0.71)	0.29 (0.27-0.32)
Year 1990	1.45 (1.32-1.60)	1.22 (1.13-1.33)	1.10 (1.02-1.19)	0.76 (0.70-0.83)	0.29 (0.27-0.31)
Year 1999	1.67 (1.46-1.91)	1.42 (1.27-1.59)	1.34 (1.20-1.50)	0.94 (0.83-1.07)	0.30 (0.27-0.34)

Model based estimates of median survival time (years) of "uncured" from flexible parametric cure model and non-mixture cure model

### Proportional excess hazards model

Table 3 shows parameter estimates from 3 flexible parametric survival models. All models include age and calendar periods as categorical variables and model the baseline log cumulative excess hazard with knots at centiles 0, 25, 50, 75, 95 and the last 12 years from diagnosis. We chose to put the last knot outside the range of our data to make sure that we don't impose a cure point too early. Even though we don't assume the cumulative excess hazard to be constant within the data, the restriction will still have some implications on the gradient of the cumulative hazard before the last knot. The first model in Table 3 is a standard flexible parametric survival model with no time-dependent effects, i.e. a proportional excess hazards model. The spline variables in model 1 are calculated backwards as described in equation (12), to make the intercept comparable to a flexible parametric cure model. The excess hazard ratios and the fitted values are the same as when splines are calculated in standard way (not shown). Model 2 is a flexible parametric cure model with no time-dependent effects, so the only difference between model 1 and 2 is the restriction of a constant cumulative excess hazard from the last knot. The intercept in both model 1 and 2 are interpreted as the log cumulative excess hazard at the last knot, and since both models are proportional excess hazards models the parameter estimates for the covariates are interpreted as log excess hazards ratios. We have omitted the parameters for the spline parameters. The estimates from model 1 and 2 are very similar. Since the cure model is nested within the standard flexible parametric survival model, a likelihood ratio test comparing the two models can be used to test if the extra parameter (the linear spline term set to zero in the cure model) in the standard model is significant. A significant result then suggests that we can reject that the linear spline term is zero, meaning that cure is not reached. From the life table estimates of relative survival we see that the relative survival seems to reach a plateau. This shows how hard it is to rely on formal tests for assessing the assumption of cure, especially in large datasets where comparisons between models are often significant. Even if the standard flexible parametric model gives a better fit to the data we are, in situations like this when the differences are in practice small, willing to give up some model fit to gain the informative estimates that the cure model gives.

**Table 3:** Parameter estimates from flexible parametric survival models

Parameter	Standard FPM splines calculated backwards	Restricted FPM incorporating a cure proportion	Restricted FPM including time-dependent effects
Intercept	0.385 (0.032)	0.350 (0.031)	0.279 (0.033)
Age group			
*<*50	-	-	-
50-59	0.089 (0.034)	0.089 (0.034)	0.085 (0.035)
60-69	0.214 (0.030)	0.212 (0.031)	0.184 (0.031)
70-79	0.385 (0.030)	0.382 (0.030)	0.284 (0.031)
80+	0.788 (0.032)	0.785 (0.032)	0.532 (0.035)
LRT	p *<*0.0001	p *<*0.0001	p *<*0.0001
Calendar period			
1953-1964	-	-	-
1965-1974	-0.339 (0.029)	-0.340 (0.029)	-0.264 (0.032)
1975-1984	-0.741 (0.028)	-0.743 (0.028)	-0.585 (0.030)
1985-1994	-0.922 (0.027)	-0.924 (0.027)	-0.769 (0.029)
1995-2003	-1.190 (0.027)	-1.194 (0.027)	-1.025 (0.030)
LRT	p *<*0.0001	p *<*0.0001	p *<*0.0001
LRT comparing to the previous model		p *<*0.0001	p *<*0.0001
AIC	92285.22	92323.95	91590.12
BIC	92398.63	92429.26	91825.04

Parameter estimates (standard errors) from flexible parametric survival models

FPM, flexible parametric survival model; LRT, likelihood ratio test; AIC, Akaike's information criterion; BIC, Bayesian information criterion.

The third model in Table 3 is a flexible parametric cure model that includes time-dependent effects for both age group and calendar periods. We only present the parameters for the constant effects in Table 3. The model parameters are harder to interpret, since they are no longer log excess hazard ratios. In both model 2 and 3 the parameters are transformations of cure. It has previously been shown for non-mixture cure models where a Weibull distribution is used that modelling of both Weibull parameters can be crucial [5]. Similarly we believe that time-dependent effects should usually be included in the flexible parametric cure model for most cancers.

### An example of a high cure proportion

**Figure 5:**
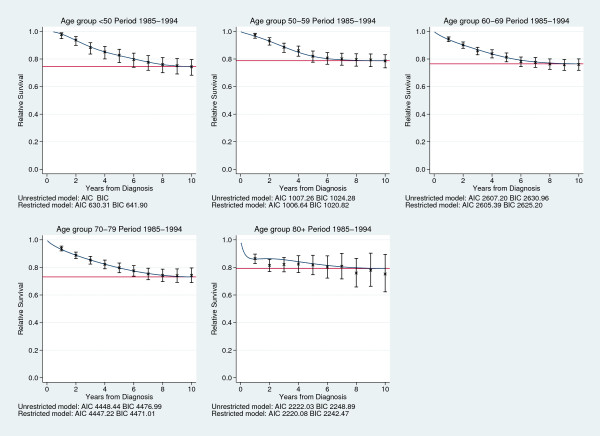
**An example of a high cure proportion**. Predicted survival and cure proportion (%) for localised cancer from flexible parametric cure models, compared to life table estimates of relative survival.

To investigate how the flexible parametric cure model performs when survival is high we restricted to localised cancer and compared, separately for each combination of age group and period, the predicted survival from the flexible parametric cure model with Ederer II life table estimates and the predicted survival from a non-mixture cure model using a Weibull distribution, in the same way as described previously for the whole cohort. The non-mixture model did not converge for all combinations of age and calendar period, mostly where the survival was high and is therefore not included here. In these scenarios the flexible parametric cure model converged and gave sensible estimates, although data were sparse when split by age and calendar period. Figure 5 shows results from flexible parametric cure models with knots at centiles 0, 25, 50, 75, 95 and the last 12 years from diagnosis, together with life table estimates, for all age groups in calendar period 1985-1994. All flexible parametric cure models were also compared to standard flexible parametric survival models using AIC and BIC, as described previously. In Figure 5 the AIC and BIC from the models can be found, the unrestricted model did not converge for the youngest age group so AIC and BIC is missing. For most models the restricted model gives a better fit, indicating that the final term in the unrestricted model is probably close to zero and that cure is reached.

## Discussion

The cure proportion is an important and interesting measure of cancer patient survival. Many of the cure models used for population-based cancer survival today rely on finding a parametric distribution flexible enough to capture the shape of the survival function, which in some scenarios is difficult to do. We here present a flexible parametric cure model, which is an extension of the flexible parametric survival model. This new method gives similar results to the Weibull non-mixture cure model, when it is reliable, and better fit when the Weibull non-mixture cure model gives biased estimates. This is illustrated here for the oldest age group where the Weibull non-mixture model gives biased estimates, but the flexible parametric survival model fits the data well.

Since the flexible parametric cure model uses splines to model the underlying survival, it is important that the model is not overly sensitive to the location of the knots. We have investigated the sensitivity and the model seems to be fairly robust to the number and location of the knots, but some care needs be taken regarding the location of the last knot. The cure proportion is estimated from the cumulative excess hazard at the last knot, so it is important not to place the last knot too early, but preferably at the last observed death time or later. It is also good to make sure that the knots are distributed along the whole follow-up time, since the model needs to fit well at the end of the follow-up, even if most of the events are at the beginning.

The mixture and non-mixture cure models are sometimes used in situations when cure is not reached within the available follow-up time in the data. This can be done since the models estimate an asymptote for the relative survival function, but estimates of cure can be very sensitive to the parametric distribution chosen. We do not recommend extrapolation in this way when using the flexible parametric cure model since the point of cure has to be chosen. Even though the position of the last knot can be outside the data the cure point should be reached within the available follow-up time.

As with other cure models, the flexible parametric cure model will give an estimate of the cure proportion even when cure is not reasonable. It is therefore important to always compare results from cure models with standard methods for relative survival and to make sure that there seem to be a proportion of patients that are cured (see Figure 2). This is not a specific drawback for the flexible parametric cure model, but for cure models in general. In contrast to the mixture and non-mixture cure model, it is for the flexible parametric cure model possible to informally test the assumption of a cure proportion since it is a restricted standard flexible parametric survival model. But these tests should be interpreted with some caution, since the comparison is based on the fit over the whole time-scale and not just towards the end where the cure proportion is estimated.

We have presented the flexible parametric cure model within a relative survival setting, since that is the method of choice for population-based studies. However, the flexible parametric survival model and the flexible parametric cure model can also be used for non-relative survival data. For example when cause of death is known and reliable, or when the background mortality is very low which is the case for childhood cancer.

To enable application of the method we have updated the Stata command for flexible parametric survival models [15], and added an option that will fitflexible parametric cure models.

## Conclusions

Cure models within the framework of flexible parametric models enables cure modelling when standard models are not flexible enough. These flexible cure models enable inclusion of older age groups and can give stage-specific estimates, which is not always possible from standard methods.

## Competing interests

The authors declare that they have no competing interests.

## Authors' contributions

PCL and TMLA concieved the project. TMLA carried out the analysis and extended the software to enable usage of the method. TMLA, PWD, SE and PCL participated in interpretation of the study results. TMLA drafted the paper, which was later revised by all co-authors through substantial contributions to the contents of the paper. All authors read and approved the final manuscript.

## Pre-publication history

The pre-publication history for this paper can be accessed here:

http://www.biomedcentral.com/1471-2288/11/96/prepub
